# Integrating delivery systems and microenvironmental cues to accelerate clinical translation of cardiac reprogramming

**DOI:** 10.1016/j.mtbio.2026.103529

**Published:** 2026-08-04

**Authors:** Boram Son, Gwang Yeol Park, Ildoo Jeong, Hwanhui Kim, Jinkyu Lee, Jinmyoung Joo, Wonhwa Lee, Hee Ho Park

**Affiliations:** a Department of Integrative Biotechnology, Kookmin University, Seoul, 02707, South Korea; b Department of Biotechnology, College of Life Sciences and Biotechnology, Korea University, Seoul, 02841, South Korea; c SIMPLE Planet Inc., Seoul, 08390, South Korea; d School of Chemical and Biological Engineering, Seoul National University, Seoul, 08826, South Korea; e Department of Biomedical Engineering, Ulsan National Institute of Science and Technology, Ulsan, 44919, South Korea; f Department of Chemistry, Sungkyunkwan University, Suwon, 16419, South Korea

**Keywords:** Direct cardiac reprogramming, Direct conversion, Cardiac regeneration, Cardiomyocyte, Heart failure treatment

## Abstract

Direct cardiac reprogramming, which converts cardiac fibroblasts into functional cardiomyocytes, has emerged as a promising regenerative strategy for heart failure. Preclinical studies have demonstrated its potential to mitigate adverse remodeling; however, critical challenges remain in delivery precision, reprogramming efficiency, and scalability for clinical translation. Here, we review innovations in delivery systems and microenvironmental engineering aimed at overcoming these barriers. Integration-free viral vectors (e.g. Sendai virus and adeno-associated virus 5) and non-viral platforms (e.g. nanoparticles and modified ribonucleic acid) have shown improved targeting of cardiac fibroblasts and enabled transient reprogramming. In parallel, microenvironmental modulation—such as immune regulation, biomechanical tuning of substrate stiffness, and the use of 3D-bioprinted matrices—has enhanced the survival and functionality of reprogrammed cells. Recent studies emphasize the need to balance genomic safety with delivery efficiency. While viral systems can achieve sustained factor expression, non-viral approaches minimize integration-associated risks. Large-animal models have further demonstrated the role of immune modulation and tissue-specific hydrogels in improving therapeutic retention. Advances in pericardial delivery systems and optimized manufacturing protocols now pave the way for future clinical translation. Nonetheless, unresolved issues—such as the long-term genomic stability of reprogrammed human cells and immune compatibility—still warrant rigorous investigation. By integrating targeted delivery, microenvironmental engineering, and scalable production, direct reprogramming strategies could redefine heart failure therapy. Collaborative efforts to address residual scientific and technical hurdles will determine their potential to benefit the 64 million patients affected globally.

## Highlights

• Direct cardiac reprogramming converts fibroblasts into functional cardiomyocytes to treat heart failure.
• Recent advances in delivery systems and microenvironmental engineering have improved reprogramming efficiency and therapy.
• Integration-free viral vectors and nanoparticle-based systems enhance safety and targeting precision.
• Microenvironmental modulation, including immune regulation and 3D-bioprinted matrices, promotes cell survival and maturation.
• Clinical translation advances via optimized delivery, but long-term genomic stability and immune safety need validation.

## Graphical abstract

**Figure:**
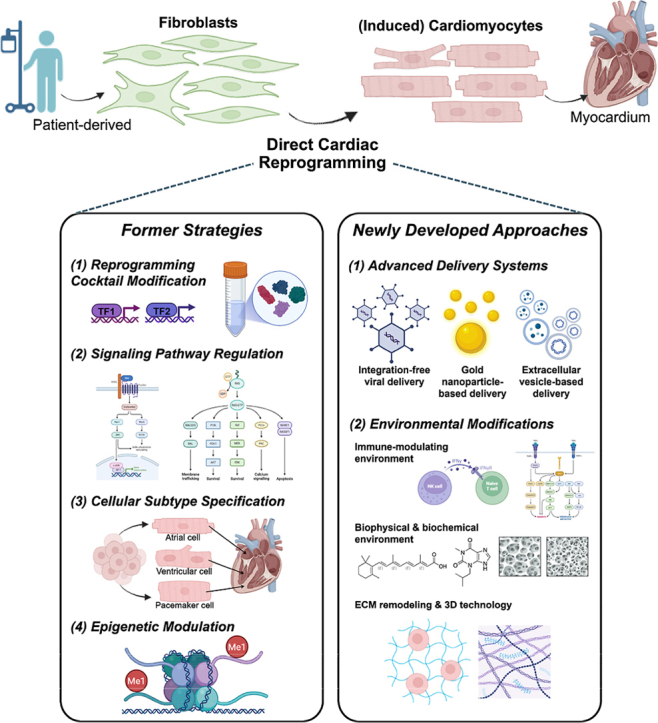

## 1 Introduction

Cardiovascular diseases (CVDs) remain the leading cause of death worldwide, accounting for approximately 17.9 million lives lost each year—a staggering figure that represents over 30% of all global deaths and translates to an average of 56,000 lives claimed each day [1]. Within this broad epidemiological landscape, heart failure represents the major end-stage clinical sequelae and a primary global health burden, currently affecting over 64 million patients worldwide [2]. While heart failure essentially manifests when the heart fails to pump a sufficient amount of blood to meet the metabolic demands of the entire body, its prevalence continues to escalate rapidly alongside rising global trends in heart and circulatory conditions [3]. Heart failure is most commonly caused by ischemic heart disease–associated injury, chemotherapy-induced cardiotoxicity, and various cardiomyopathies [4,5].

Advances in modern medicine have yielded various options for heart failure treatment. Pharmacological therapies include drugs that lower blood pressure or blood volume, as well as agents that increase cardiac contractility [6]. However, these agents do not restore damaged heart tissue—the fundamental cause of heart failure—but instead focus on alleviating symptoms, offering only temporary benefits. Another treatment option is heart transplantation, with or without mechanical assist devices [7]. Because auxiliary devices alone cannot fully replicate the function of an intact heart, transplantation of a donor organ remains essential for definitive treatment. Yet the scarcity of donor hearts remains a major limitation [8]. Thus, there is an urgent need for therapies that can restore the function of damaged heart tissue.

In this context, cell-based therapies for regenerating damaged myocardium have gained increasing attention. Among the four major cardiac cell types—cardiomyocytes (CMs), cardiac fibroblasts (CFs), smooth muscle cells (SMCs), and endothelial cells (ECs)—CMs are essential for myocardial function and are the primary contributors to cardiac regeneration [7,9,10]. However, adult CMs lack the capacity for self-renewal. Consequently, lost CMs are replaced by fibroblasts, leading to fibrosis and ultimately heart failure [11,12]. To address this, cells with cardiomyogenic potential have been transplanted into the injured myocardium as a therapeutic approach [13]. Several cell sources have been explored for generating new CMs, including embryonic stem cells (ESCs), induced pluripotent stem cells (iPSCs), mesenchymal stem cells (MSCs), and cardiac progenitor cells [6,[14], [15], [16], [17]]. Despite their differentiation potential, these sources present challenges in cell fate regulation and carry a risk of tumorigenesis [18].

To overcome these limitations, direct cardiac reprogramming was first reported in 2010 as a strategy for cardiac regeneration [19]. Various genetic and cellular engineering techniques have since been developed to directly induce CMs. However, engraftment of terminally differentiated cells into injured myocardium has been inefficient, resulting in low survival rates of transplanted cells [20]. Recent innovations suggest that cell-free delivery of reprogramming factors can induce *in vivo* reprogramming and improve cardiac recovery [21,22].

Over the past 15 years, the field of direct cardiac reprogramming has undergone a dramatic maturation, evolving from an academic proof-of-concept into a tangible translational framework [23]. Since the landmark discovery of the core Gata4, Mef2c, and Tbx5 (GMT) transcription factor pool in 2010, publication trends have shifted exponentially from descriptive *in vitro* screens toward high-density preclinical validations [19]. This academic expansion has been accompanied by a parallel surge in global patent activities focusing on multi-cistronic stoichiometric vectors, organ-targeted lipid nanoparticles, and small-molecule chemical cocktails [[24], [25], [26]]. Emerging commercial efforts and spin-off biotech ventures are actively standardizing clinically compliant current good manufacturing practice (cGMP) manufacturing platforms to advance cell-free direct conversion therapies toward early-phase clinical pipelines [27]. This industrial pivot underscores the critical requirement to bridge fundamental matrix bioengineering with precision factor delivery frameworks to overcome long-standing efficiency and maturation barriers [28,29].

**Fig. 1:**
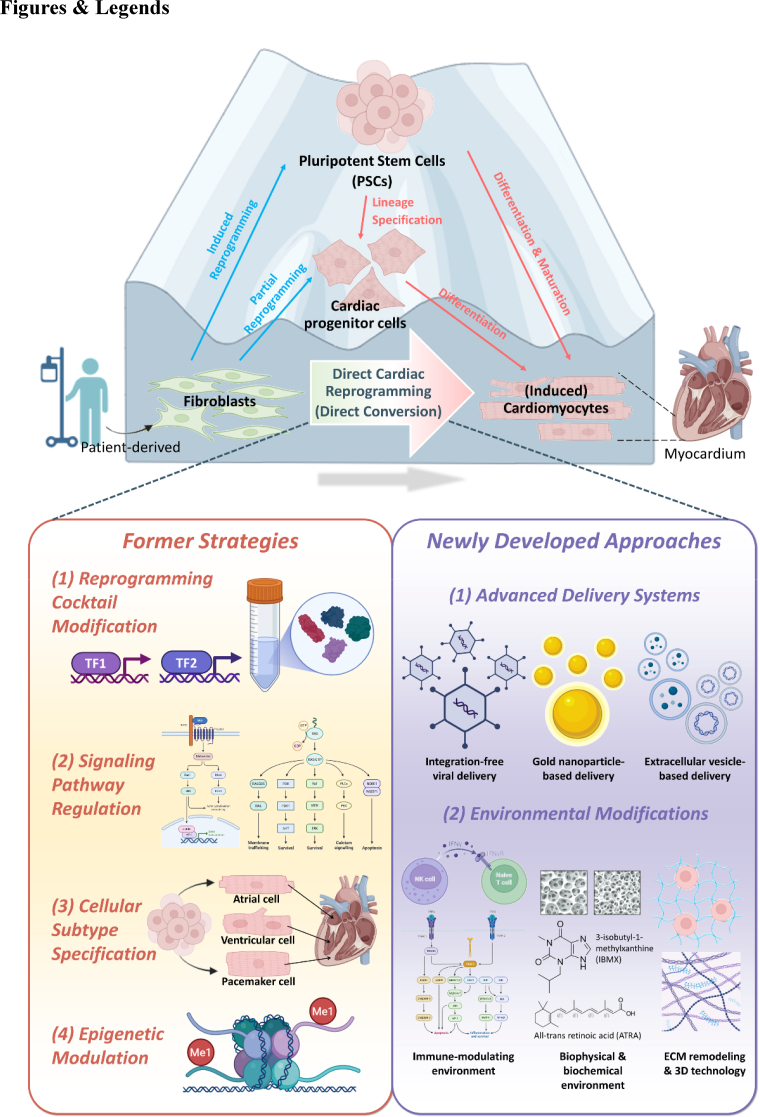
Schematics of cardiac regeneration and direct cardiac reprogramming strategies. Cardiomyocytes (CMs), the essential muscle cells of the heart, are the primary focus for regeneration following cardiac injury or disease. The conventional approach involves differentiating pluripotent stem cells (PSCs) into cardiac progenitor cells, which then mature into functional induced CMs. Recent advances have enabled the direct conversion of patient-derived somatic cells (e.g., fibroblasts) into CMs, bypassing the PSC stage. These direct cardiac reprogramming methods offer promising avenues for cardiac regeneration. This paper categorizes these approaches into two main groups: Former Strategies (orange, left) and Newly Developed Approaches (purple, right). Former Strategies include: (1) reprogramming cocktail modification; (2) signaling pathway regulation; (3) cellular subtype specification; and (4) epigenetic modulation. Newly Developed Approaches include: (1) advanced delivery systems, and (2) environmental modifications. (For interpretation of the references to colour in this figure legend, the reader is referred to the Web version of this article.)

Here, we introduce diverse approaches for advancing direct cardiac reprogramming (Fig. 1). Conventional strategies to improve reprogramming efficiency include modification of reprogramming cocktails, activation or inhibition of signaling pathways, specification of cellular subtypes, and epigenetic modulation. In addition, emerging approaches—such as delivery system optimization and microenvironmental modulation—have shown promise in enhancing direct cardiac reprogramming and improving heart failure outcomes. This review summarizes the historical development of direct cardiac reprogramming and highlights recent advancements aimed at maximizing its therapeutic potential. To provide a clear overview for the readers, we first dissect the chronological optimization of core genetic and signaling cocktails in section 2, followed by a critical cross-examination of integration-free viral and advanced non-viral delivery platforms in section 3. Section 4 explores advanced tissue-engineering strategies designed to modulate the biophysical and immune microenvironment. Finally, section 5 outlines a comprehensive preclinical roadmap, addressing cGMP bottlenecks and electrical compatibility required for successful patient clinical entry.

## 2 Former strategies to improve direct cardiac reprogramming efficiency

### 2.1 Reprogramming cocktail modification

**Fig. 2:**
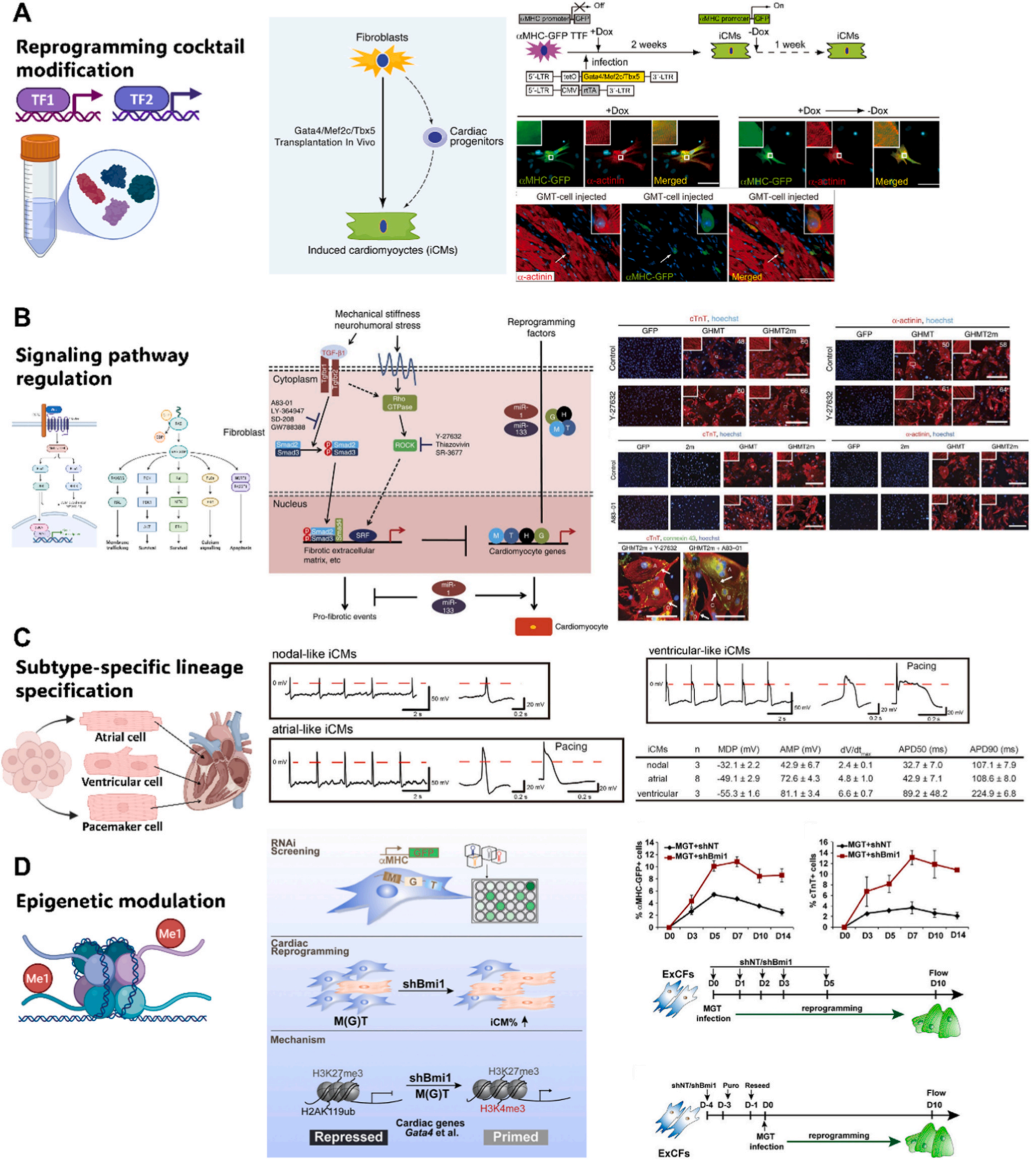
Established strategies for enhancing cardiac reprogramming efficiency. (A) Reprogramming cocktail optimization. Fibroblasts are directly reprogrammed into induced cardiomyocytes (iCMs) using three transcription factors (Gata4, Mef2c, Tbx5; GMT). *In vivo* reprogramming of transplanted cardiac fibroblasts transduced with GMT confirms functional conversion. Data are obtained and reused from Ieda et al., Direct reprogramming of fibroblasts into functional cardiomyocytes by defined factors. *Cell.* 2010, 142, 375–386 with permission. (B) Signaling pathway modulation. Suppression of pro-fibrotic signaling (TGF-β/ROCK pathways) via inhibitors significantly enhances iCM reprogramming efficiency and functional maturation. Data are obtained and reused from Zhao et al., High-efficiency reprogramming of fibroblasts into cardiomyocytes requires suppression of pro-fibrotic signaling. *Nat. Commun.* 2015, 6, 8243 under a Creative Commons Attribution 4.0 International License with permission. (C) Subtype-specific lineage specification. Sendai virus-delivered GMT (SeV-GMT) generates functionally distinct iCM subtypes: nodal-like, atrial-like, and ventricular-like cardiomyocytes. Data are obtained and reused from Miyamoto et al., Direct *in vivo* reprogramming with sendai virus vectors improves cardiac function after myocardial infarction. *Cell Stem Cell*. 2018, 22(1), 91–103 e5 with permission. (D) Epigenetic regulation. Bmi1 downregulation markedly improves mouse fibroblast-to-iCM conversion. Bmi1 depletion functionally substitutes for Gata4 during early reprogramming stages. Data are obtained and reused from Zhou et al., Bmi1 is a key epigenetic barrier to direct cardiac reprogramming. *Cell Stem Cell.* 2016, 18, 382–395 with permission.

In 2010, Ieda et al. reported a promising therapeutic avenue with the discovery that CFs could be directly reprogrammed into functional CMs, bypassing the need for an intermediate iPSC stage (Fig. 2A) [19]. This breakthrough was enabled by the identification of three key transcription factors (GMT) that initiated the reprogramming process. The initial identification of GMT as a transcription factor combination capable of converting CFs into CM-like cells marked a milestone in the field. Introducing GMT resulted in approximately 17% of fibroblasts transforming into green fluorescence protein (GFP)-positive cells. Although significant, the efficiency of direct cardiac reprogramming remained low, prompting further efforts to optimize the reprogramming cocktail for improved outcomes.

Subsequent studies focused on enhancing GMT to boost reprogramming efficiency. In 2012, Song et al. added Hand2 to the GMT combination, creating GMT with Hand2 (GHMT) [30]. This modification substantially improved efficiency, with approximately 9.2% of tail-tip fibroblasts and 6.8% of adult CFs expressing CM markers. Moreover, these induced CMs (iCMs) exhibited spontaneous beating—a critical feature for functional integration. In 2013, Hirai et al. further improved the process by increasing the transcriptional activity of Mef2c within the GHMT cocktail [31]. By fusing MyoD transactivation domains to GHMT factors, they achieved a 15-fold increase in the beating efficiency of iCMs compared with fibroblasts transduced with wild-type Mef2c. Around the same time, Addis et al. reported significant gains by adding NKX2.5 and Hand2 to the GMT cocktail [32]. Delivered via doxycycline (Dox)-inducible lentiviral vectors, this modification increased reprogramming efficiency by 50-fold compared with GMT alone, producing iCMs with typical cardiac markers, spontaneous beating, and robust calcium oscillations.

Recognizing the importance of optimizing reprogramming factor stoichiometry, Wang et al. examined polycistronic retroviral constructs encoding different arrangements of G, M, and T to modulate expression levels [33]. Two constructs with high Mef2c and low Gata4 and Tbx5 levels (MGT and MTG) achieved superior cardiac reprogramming efficiency compared with conventional mixed-vector approaches. Building on these findings, Ma et al. extended the strategy to *in vivo* applications [34]. In a myocardial infarction (MI) model, polycistronic MGT vectors yielded higher iCM numbers, well-formed sarcomeric structures, improved functional recovery, and reduced scar formation compared with conventional GMT vectors.

To facilitate instant scannability of these foundational advances, the chronological development and key functional features of these optimized genetic cocktails are systematically summarized in Table 1. Crucially, as highlighted by these milestones, recent structural insights indicate that direct reprogramming efficiency is dictated not merely by the empirical presence of these transcription factors, but precisely by their stoichiometric expression ratio, which governs global chromatin accessibility and downstream transcriptional cascades [33,35,36]. Biochemical evaluations utilizing polycistronic retroviral vector configurations—such as the MGT and MTG arrangements developed by Wang et al.—demonstrate that a high-Mef2c and low-Gata4/Tbx5 stoichiometric balance optimizes both the conversion efficiency and the functional quality of generated iCMs [33].

**Table 1:** Chronological optimization of core genetic reprogramming cocktails.

Core cocktail	Delivery platform/key modifications	Reprogramming efficiency/key structural features	Ref
**GMT**	Retroviral individual vectors	∼17% αMHC-GFP^+^ cells; baseline lineage conversion	[19]
**GHMT**	Retroviral individual vectors	9.2% TTFs/6.8% adult CF markers; spontaneous beating	[30]
**Mef2c-MyoD fusions**	Retroviral transactivation domain engineering	15-fold increase in functional beating iCM generations	[31]
**GMTHN**	Dox-inducible polycistronic lentiviral systems	50-fold efficiency gain over baseline GMT; robust calcium oscillations	[32]
**MGT (high-M)**	Polycistronic single-vector stoichiometry	Optimal structural sarcomeric assembly and high fidelity *in vitro*	[33]
**MGT (*in vivo*)**	Intramyocardial polycistronic viral delivery	Significant scar reduction and functional mechanical recovery post-MI	[34]

On a molecular level, Mef2c operates as a rate-limiting pioneer factor in human and rodent cardiac reprogramming [23]. When overexpressed relative to Gata4 and Tbx5, Mef2c synergistically binds to closed heterochromatic cardiogenic loci, and recruits histone acetyltransferases like p300, thereby enriching active H3K27ac marks at core cardiac super-enhancers [37]. This high-Mef2c stoichiometry effectively suppresses the competing, pro-fibrotic fibroblast gene programs that are otherwise reactivated when Gata4 is in excess [38]. Furthermore, precise stoichiometric control prevents the molecular squelching of shared transcriptional co-activators, facilitating the high-affinity cooperative assembly of the physical Gata4/Mef2c/Tbx5 ternary complex on downstream sarcomeric promoters, including Myh6 and Tnnt2 [33,39]. Building on these mechanistic principles, Ma et al. extended this single polycistronic MGT configuration to *in vivo* MI models, verifying that such stoichiometric optimization yields a significantly higher density of well-formed sarcomeric structures, superior regional functional recovery, and reduced fibrotic scar volume compared to conventional mixed-vector pools [34]. Collectively, these findings confirm that precise stoichiometric orchestration over epigenetic remodeling is a prerequisite for clinical-grade human translation.

### 2.2 Signaling pathway regulation

While significant progress has been made in direct cardiac reprogramming, challenges remain—notably the need to optimize the efficiency and quality of iCM generation. Researchers have focused on manipulating signaling pathways to address these limitations. One of the major fibroblast-associated pathways is transforming growth factor-β (TGF-β). Inhibition of TGF-β has shown promise in enhancing cardiac reprogramming. In 2014, Ifkovits et al. reported that blocking TGF-β with SB431542 increased iCM generation efficiency in both mouse embryonic fibroblasts and adult fibroblasts [40]. The addition of SB431542 to the transcription factor combination GMT + Hand2 and NKX2.5 produced a fivefold increase in iCM yield. In 2015, Zhao et al. demonstrated that simultaneous suppression of TGF-β and Rho-associated kinase (ROCK) pathways markedly improved efficiency, converting up to 60% of adult fibroblasts into beating iCMs (Fig. 2B) [41]. In 2017, Mohamed et al. combined TGF-β and Wnt pathway inhibition with the GMT cocktail, achieving an eightfold increase in reprogramming efficiency from CFs [42]. Their research achieved an 8-fold improvement in reprogramming efficiency from CFs. This study demonstrated that Wnt pathway inhibition is a key driver of cardiac reprogramming.

Notch pathway inhibition has similarly emerged as a strategy to enhance reprogramming [43]. In 2017, Abad et al. showed that DAPT, a Notch inhibitor, improved the conversion of mouse fibroblasts into iCMs using the GHMT cocktail [44]. This treatment yielded a more mature CM phenotype with elevated Akt1 expression and enhanced binding of Mef2c to cardiac structural gene promoters, thereby increasing transcriptional activity. The importance of Akt1 in direct cardiac reprogramming was further emphasized in 2015 by Zhou et al. who found that adding Akt1 to the GHMT combination boosted reprogramming efficiency and promoted iCM maturation [45]. Their work identified Akt/protein kinase B as a critical accelerator of the reprogramming process.

Beyond these developmental pathways, immune responses have emerged as important regulators of cardiac reprogramming [[46], [47], [48]]. Studies have shown that immune signaling is required for successful reprogramming, as knockdown of immune regulatory genes reduces efficiency [49]. Intriguingly, while immune responses are necessary for human cardiac reprogramming, inhibition of inflammatory pathways has been found to enhance mouse cardiac reprogramming [50]. This cross-species divergence is fundamentally driven by distinct cytokine cascades and immune cell subtype behaviors that dictate downstream epigenetic accessibility. In human direct cardiac reprogramming, transient activation of innate immune signaling—specifically via the Toll-like receptor 3 (TLR3) pathway—is indispensable for prompting the nuclear translocation of NF-κB. This TLR3–NF-κB axis upregulates critical downstream target cascades, including prostaglandin-endoperoxide synthase 2 (PTGS2/COX-2), which temporarily relaxes the highly restrictive human heterochromatin architecture, thereby allowing exogenous transcription factors to successfully gain access to tightly sealed cardiogenic super-enhancers [51]. Conversely, in murine systems, acute and chronic inflammation acts as a severe institutional barrier to lineage conversion. In rodent post-injury microenvironments, persistent infiltration of host CCR2^+^ inflammatory M1 macrophages establishes a hostile circuit dominated by the interferon-α/β receptor (IFN-IFNAR) axis [52]. Activation of this IFN-IFNAR cascade drives the hyper-phosphorylation of STAT1 (p-STAT1), which actively suppresses the transcription of essential cardiac programming factors such as Gata4 and Mef2c [53]. Consequently, human transdifferentiation requires targeted, brief innate pathways to unlock chromatin barriers, whereas murine systems necessitate the timely application of anti-inflammatory compounds or chemokine receptor inhibitors to shield converted cells from M1-macrophage-mediated transcriptional suppression. This species-specific immune variance underlines the necessity of aligning front-end cell mechanisms with the localized immune cell dynamics discussed in section 4.1. However, manipulating these developmental and immunological pathways frequently introduces conflicting perspectives within the literature, highlighting the contextual complexity of direct conversion. A prime example resides in Wnt signaling pathway regulation; while numerous studies demonstrate that Wnt pathway inhibition post-transduction is a key driver for accelerating rodent and human cardiac reprogramming [42], counter-reports indicate that transient Wnt activation during the absolute initial stage of factor introduction is required to prime fibroblast plasticity. Such temporal contradictions prove that small-molecule-mediated signaling regulation cannot be treated as a static, continuous block, but instead demands rigorous chronological orchestration.

### 2.3 Subtype-specific lineage specification strategies

The heart is a remarkably intricate organ composed of diverse cell types, including distinct CM subtypes—atrial, ventricular, and pacemaker cells—each with unique anatomical locations, gene expression profiles, and electrophysiological properties [54,55]. Together, these subtypes ensure the precise orchestration of cardiac function. Subtype specificity is critical, as each CM subtype plays a distinct role in maintaining effective blood circulation. The sinoatrial node, the heart's primary pacemaker, initiates the cardiac impulse, depolarizing atrial CMs [56,57]. The atrioventricular node and the conduction system then relay this impulse to ventricular CMs, triggering ventricular contraction [58]. Given these distinct functions, the loss or dysfunction of any subtype can lead to specific forms of heart disease. For example, MI primarily affects ventricular CMs while sparing atrial and pacemaker cells [59,60]. Consequently, for *in vivo* cardiac reprogramming to be therapeutically effective, reprogramming strategies must transcend non-specific cellular conversion and actively enforce targeted ventricular specification within the infarcted ventricular myocardium to avoid lethal conduction abnormalities or ectopic pacing.

Although the clinical importance of subtype specification is clear, early methodological approaches achieved only limited, stochastic lineage steering. Nam et al. examined this aspect using fibroblasts from Hcn4-GFP reporter mice [61]. They found that *in vitro* reprogramming with a GHMT-based cocktail could induce all three major CM subtypes—atrial, ventricular, and pacemaker cells. The reprogrammed cells displayed electrophysiological properties consistent with their respective subtypes, as defined by intracellular action potentials. In other words, GHMT-mediated reprogramming produced a mixed population of CM phenotypes. Similarly, Miyamoto et al. reported that GMT transduction generated heterogeneous iCM subtypes (Fig. 2C) [62]. To resolve this structural disconnection and transform subtype generation from an empirical phenomenon into a controllable strategy, recent frontiers have focused on manipulating the transcriptional and microenvironmental gating of subtype identity [36]. Accumulating studies demonstrate that modulating the core stoichiometry of genetic cocktails or incorporating downstream signaling blockades acts as an instructive strategy to bias cell fate; for instance, supplementing the GMT baseline with precise factors like Hand2 or optimizing high-Mef2c packaging specifically enriches the ventricular-like gene network while suppressing pacemaker-associated hyperpolarization-activated cyclic nucleotide-gated channels [33,38,39]. Furthermore, as discussed in section 4, synchronizing these genetic cocktails with targeted biophysical cues—such as mechanical stiffness matching native ventricular tissue (∼10 kPa) or microgroove-mediated alignment—serves as an active bioengineering strategy to drive ventricular-specific structural and electrophysiological maturation [63,64]. Determining the degree of such strategic ventricular steering achieved in *in vivo*-generated iCMs will be critical for clinical translation, ensuring that reprogrammed cells can replace lost ventricular CMs after MI and contribute to functional recovery [65,66].

### 2.4 Epigenetic modulation

Since epigenetic factors play crucial roles in determining cell fate, their manipulation can enhance the efficiency of direct cardiac reprogramming. Deoxy ribonucleic acid (DNA) methylation, a fundamental epigenetic modification, regulates gene transcription and cellular differentiation [67]. Notably, B cell–specific Moloney murine leukemia virus integration site 1 (Bmi1), an epigenetic regulator associated with DNA methylation, has emerged as a critical barrier to cardiac reprogramming (Fig. 2D) [68]. Screening studies have shown that reduced Bmi1 expression correlates with increased levels of the active histone mark H3K4me3 and decreased levels of the repressive mark H2AK119ub at cardiogenic loci. Consequently, Bmi1 suppression enhances the generation of beating iCMs, highlighting the importance of DNA methylation–associated epigenetic changes in reprogramming.

Micro ribonucleic acid (miRNAs), another key class of gene expression regulators, have also been leveraged to improve cardiac reprogramming [69,70]. Combining a Janus kinase (JAK) inhibitor with muscle-specific miRNAs—such as miR-1, miR-133, miR-208, and miR-499—proved effective in generating iCMs from CFs [71]. Among these, miR-133 supplementation significantly increased reprogramming efficiency [72]. Because miR-133 targets the zinc finger protein Snai1, a regulator of epithelial-to-mesenchymal transition, its addition accelerated fibroblast reprogramming [73]. These miRNA-based strategies thus represent a promising approach for enhancing direct cardiac reprogramming [74].

Chromatin-remodeling factors have also been employed for epigenetic modulation, exemplified by Baf60c, a cardiac-specific subunit of the SWItch/Sucrose Non-Fermentable (SWI/SNF) complex [75]. These factors initiate a cascade of epigenetic modifications that facilitate reprogramming. In addition, histone readers such as PHD finger protein 7 (PHF7)—which specifically bind to histone H3K4me3 and H3K4me2 marks—can further enhance chromatin accessibility [76]. PHF7 interacts with the SWI/SNF complex, promoting its recruitment to cardiac super-enhancers and facilitating chromatin remodeling [77]. Collectively, these findings demonstrate the central role of chromatin remodeling in cardiac reprogramming.

### 2.5 Methodological and physiological stages: distinguishing rodent, large-animal, and human models

To preserve the academic credibility of direct cardiac reprogramming, it is critical to implement a strict tri-layered distinction among small-animal proof-of-concept studies, large-animal preclinical validations, and human clinical applications. A vast majority of early mechanistic breakthroughs have been fundamentally established using mouse embryonic fibroblasts (MEFs) or rodent injury models [19]. While these small-animal models provide invaluable screening platforms for reprogramming cocktails, direct extrapolation of rodent success to human therapy faces steep physiological and anatomical gaps [78]. Rodent hearts possess low myocardial mass, rapid intrinsic heart rates, and high baseline cellular plasticity. In contrast, human and large-animal (such as porcine or non-human primate) hearts present a vastly different geopolitical environment, characterized by dense, ischemic post-MI scar tissue that acts as a physical barrier to vector diffusion [79]. Consequently, intermediate large-animal studies—such as those evaluating long-term safety, arrhythmogenic risks, and delivery retention via porcine MI models—serve as an indispensable translational bridge [80]. Relying solely on rodent data while omitting the rigorous transitional metrics of large-animal models overstates the immediate readiness of the technology; therefore, contextualizing these distinct experimental phases is essential for accurately evaluating the clinical feasibility of advanced delivery systems and microenvironmental strategies.

## 3 Newly developed approaches for advanced delivery system

### 3.1 Integration-free gene delivery strategies

Integration-based approaches using retroviral (RV) and lentiviral (LV) vectors have made significant contributions to *in vivo* cardiac reprogramming. RVs integrate their genetic payload into the host genome during cell division, enabling cell type–specific gene transfer [81]. LVs, closely related to RVs, offer the additional advantage of targeting both dividing and non-dividing cells, thereby broadening their applicability [82]. Although these vectors have enabled precise gene delivery to CFs, several challenges remain, including the need to optimize gene transfer efficiency, improve specificity, and address safety concerns associated with genome integration [83].

**Fig. 3:**
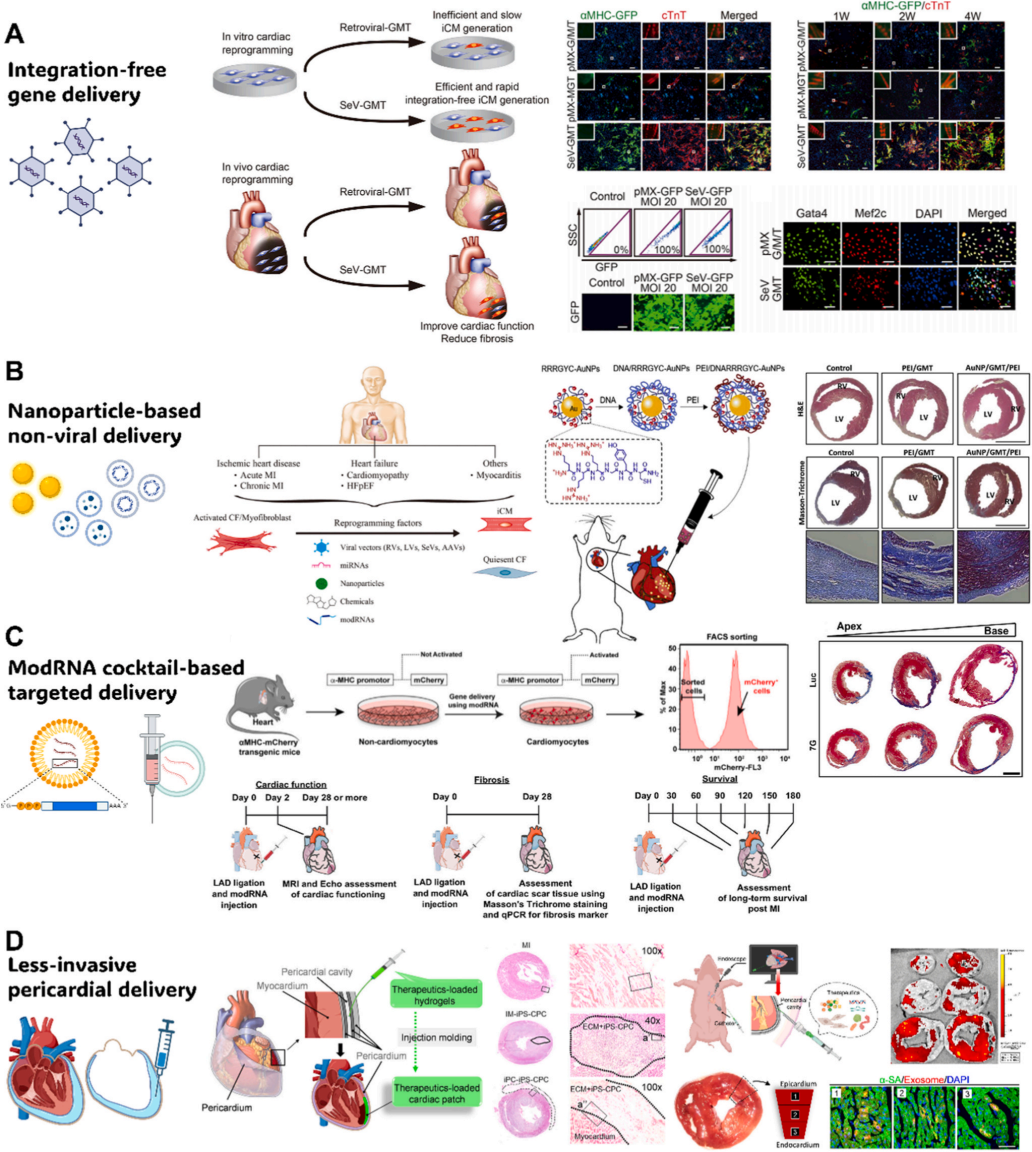
Advanced delivery systems for cardiac reprogramming. (A) Integration-free viral vector delivery. Sendai virus-mediated direct *in vivo* reprogramming enhances cardiac function post-myocardial infarction. Data are obtained and reused from Miyamoto et al., Direct *in vivo* reprogramming with sendai virus vectors improves cardiac function after myocardial infarction. *Cell Stem Cell*. 2018, 22(1), 91–103 e5 with permission. (B) Non-viral nanoparticle delivery. Gold nanoparticle-based gene delivery enables efficient fibroblast-to-cardiomyocyte reprogramming and improves cardiac function after infarction. Data are obtained and reused from Yamada et al., Development of direct cardiac reprogramming for clinical applications. *J*. *Mol*. *Cell*. *Cardiol*. 2023, 178, 1-8; Chang et al., Efficient *in vivo* direct conversion of fibroblasts into cardiomyocytes using a nanoparticle-based gene carrier. *Biomaterials*. 2019, 192, 500–509 with permission. (C) ModRNA therapy. modRNA-based delivery enhances cardiac function, reduces fibrosis, and increases survival rates. Data are obtained and reused from Kaur et al., Direct reprogramming induces vascular regeneration post muscle ischemic injury. *Mol*. *Ther*. 2021, 29(10), 3042-58 with permission. (D) Minimally invasive pericardial delivery. Hydrogel-mediated therapeutic agent delivery into the pericardial cavity promotes cardiac repair while mitigating immune responses. Data are obtained from Zhu et al., Minimally invasive delivery of therapeutic agents by hydrogel injection into the pericardial cavity for cardiac repair. *Nat*. *Commun*. 2021, 12(1), 1412 under a Creative Commons Attribution 4.0 International License with permission. (For interpretation of the references to colour in this figure legend, the reader is referred to the Web version of this article.)

By contrast, integration-free delivery methods, such as Sendai viral (SeV) vectors, offer a safer alternative for cardiac reprogramming. These vectors do not integrate into the host genome, thereby minimizing risks associated with genomic alterations [84]. SeV vectors efficiently target proliferating CFs, promoting robust reprogramming [85]. Of particular note is SeV-GMT, a polycistronic SeV vector carrying the essential reprogramming factors, which has emerged as a promising tool in this field. Preclinical studies using SeV-GMT have demonstrated improved cardiac function and reduced scar formation in murine models of MI (Fig. 3A) [62,86]. While these findings firmly establish early-stage rodent proof-of-concept, translation to human therapeutics remains constrained, as robust large-animal efficacy and long-term dosage safety profiling of SeV-mediated conversion are still far less accumulated compared to rodent data [87,88]. Remaining challenges include mitigating potential immune responses and optimizing manufacturing processes for clinical translation [89].

Adeno-associated viral (AAV) vectors represent another notable integration-free option. These vectors are valued for their low immunogenicity and non-integrating nature, making them attractive for clinical gene therapy [90]. Engineered AAV variants, such as AAV-DJ, have shown promise for efficient gene delivery to CFs, with potential applications in cardiac reprogramming [91]. Importantly, AAV-mediated direct reprogramming has successfully advanced to preclinical porcine MI models, demonstrating localized reduction in fibrotic scar volume and partial mechanical recovery; however, a strict boundary must be maintained between these initial large-animal validation results and actual human readiness, as human CFs present a significantly higher epigenetic resistance that limits AAV translation [92]. Crucially, while AAV vectors are predominantly considered non-integrating and exhibit a low frequency of genomic insertion, recent literature has noted rare genomic integration events of AAV vector genomes [90,93]. Acknowledging these rare integration events is essential for accurately evaluating the long-term safety profile of AAV-mediated cardiac reprogramming, as random insertions into the host genome can potentially prompt insertional mutagenesis or unintended oncogenic transitions within the multi-cellular myocardial niche [90]. Consequently, further investigation and continuous genomic tracing are mandatory to fully evaluate the long-term safety profiles and transgene expression dynamics associated with AAV-mediated cardiac reprogramming [93].

### 3.2 Alternative gene delivery methods

#### 3.2.1 Nanotechnology-based non-viral delivery system

The field of non-viral gene delivery is rapidly evolving, with systems based on nanoparticles or nanovesicles emerging as a promising frontier in cardiac reprogramming. Among these, cationic gold nanoparticles (AuNPs) conjugated with reprogramming factors offer an innovative, non-viral, and non-toxic delivery strategy [94]. Initial studies using AuNPs-GMT for *in vivo* reprogramming have shown considerable potential, demonstrating improved efficiency and reduced toxicity compared with traditional viral vectors (Fig. 3B) [[95], [96], [97], [98]]. The unique properties of AuNPs—including their size-dependent optical characteristics, high surface-to-volume ratio, and ease of functionalization—make them particularly attractive for gene delivery [99]. These nanoparticles can be precisely engineered to carry and protect genetic cargo, while surface modifications can enhance cellular uptake and targeting specificity. Moreover, the non-toxic nature of AuNPs addresses several safety concerns associated with viral vectors, potentially paving the way for safer clinical applications [100,101].

Building on advances in nanoscale delivery systems, engineered extracellular vesicles (EVs) have emerged as a powerful platform for non-viral gene therapy in cardiac regeneration. Recent studies show that EVs functionalized with cardiac reprogramming factors (for example, the GMT cocktail) can achieve targeted delivery while minimizing immunogenicity—a key limitation of viral vectors [102]. These nanosized vesicles, naturally derived from cell membranes, inherently possess biocompatibility and the capacity to bypass immune surveillance, enhancing their therapeutic potential. By engineering EV surfaces with cardiac-targeting peptides or fusion proteins, researchers have markedly improved cell-type specificity and uptake in damaged myocardium. Furthermore, EVs loaded with modified mRNA or microRNA exhibit superior stability and controlled release kinetics compared with synthetic nanoparticle systems, enabling sustained expression of reprogramming factors.

Concurrently, lipid nanoparticles (LNPs) have garnered significant attention as a premier non-viral platform, heavily accelerated by their clinically proven success in global messenger RNA (mRNA) vaccine deployment [103]. When coupled with modified mRNA (modRNA) encoding cardiac transcription factors, LNP-modRNA platforms offer exceptional advantages, including highly efficient cellular transfection, zero risk of integration-associated insertional mutagenesis, and transient, dose-controllable kinetics that prevent prolonged, hazardous factor overexpression [104]. To overcome the liver-dominant accumulation inherent to conventional formulations, recent milestones have introduced sophisticated engineering strategies, such as selective organ targeting (SORT) technology, which systematically tunes the internal lipid composition to re-route LNP tropism directly toward the heart [105]. Recent cardiac direct reprogramming applications have demonstrated that optimized LNP-modRNA formulations can achieve robust *in vivo* delivery, successfully turning fibrotic tissue toward functional CM lineages in post-ischemic injury settings [106,107].

Although these nanoparticle platforms show transformative potential, several challenges must be addressed before clinical translation. For AuNP-based systems, key issues include the long-term biodistribution of metallic nanoparticles and the need for finer control over surface charge to balance transfection efficiency with cellular toxicity [108,109]. EV approaches face hurdles in standardizing cargo-loading efficiency and ensuring batch-to-batch consistency, owing to the biological variability of donor cells [102]. For LNP-mRNA platforms, despite their outstanding scalability, a critical evaluation reveals pivotal limitations: their brief intracellular half-life requires repeated administration to maintain the minimum threshold of transcription factor expression needed for complete CM maturation, and elevated therapeutic doses can spark transient activation of innate pattern-recognition pathways, risking localized immunogenicity [[103], [104], [105], [106], [107]]. Furthermore, achieving precise single-cell targeting exclusively for CFs remains an unresolved obstacle, as non-selective uptake by adjacent EC or SMCs could trigger unintended off-target reprogramming cascades. Crucially, from a translational standpoint, the vast majority of these synthetic (AuNP, LNP) and biological (EV) nanotechnology milestones remain strictly confined to small-animal proof-of-concept settings. Extrapolating these nanoscale successes to large-animal systems or human applications is significantly hindered by the requirement for higher therapeutic doses, which exponentially heightens the risk of systemic off-target clearance by the reticuloendothelial system and localized acute immunogenicity. More broadly, nanotechnology-based strategies require improved targeting specificity to minimize off-target organ effects and the development of scalable, GMP-compliant manufacturing processes for synthetic and biological platforms. In addition, immune interactions remain incompletely understood: although EVs inherently evade immune clearance, their surface modifications may trigger unintended responses, whereas AuNPs can accumulate in reticuloendothelial organs and potentially cause chronic inflammation [110,111].

#### 3.2.2 ModRNA cocktail-based targeted delivery system

Novel gene-delivery strategies have emerged, attracting considerable attention from the scientific community. ModRNA cocktails, such as 7G-modRNA, represent an innovative approach with the potential to enhance cardiac function and reduce fibrosis (Fig. 3C) [23,110,112,113]. However, ongoing work is addressing the limited maturity of iCMs and investigating the potential long-term consequences of this method.

Chemical reprogramming, using small molecules such as CRFVPT and 9C, has also shown promise in generating cardiac-like iCMs [95,114]. While this approach is encouraging, further investigation is needed to determine its suitability for *in vivo* applications [115].

Precision in gene delivery is critical for both therapeutic efficacy and safety. Non-selective targeting strategies using retroviruses may lead to off-target effects. To overcome this limitation, researchers are developing promoter-based selective targeting to enable precise CF reprogramming. These efforts aim to maximize therapeutic benefit while minimizing unintended outcomes.

Crucially, evaluating the safety profile of non-viral frameworks demands a rigorous assessment of off-target reprogramming risks within the multi-cellular cardiac niche and systemic circulation. Because synthetic vehicles such as LNPs and naked modRNA cocktails inherently lack natural evolutionary tropism, their local or systemic administration carries the persistent risk of non-selective endocytosis by adjacent non-target cell types, including ECs and SMCs, or systemic accumulation in metabolic clearance organs like the liver and spleen [116]. Inadvertent delivery of potent cardiogenic transcription factors to these non-target niches could prompt partial or aberrant transdifferentiation cascades, risking severe pathological consequences such as endothelial-to-mesenchymal transitions, vascular wall destabilization, localized loss-of-function, or insertional-independent oncogenesis [117]. To mitigate these translational vulnerabilities, next-generation frontiers are shifting from non-selective encapsulation toward multi-layered precision engineering [118]. Researchers are actively integrating CF-specific promoters (e.g., Postn or Tcf21) directly into the genetic payload to restrict transcription factor translation exclusively within active myofibroblasts [119]. Concurrently, conjugate engineering utilizing fibroblast-targeting surface peptides or antibodies is being deployed to achieve high-affinity biophysical sorting, establishing a vital safeguard for non-viral clinical configurations.

#### 3.2.3 Pericardial approach-based less-invasive delivery system

Current cardiac reprogramming protocols primarily rely on direct intramyocardial injection, a method associated with substantial surgical risks and limited clinical applicability [120]. In response to the need for less invasive strategies, researchers are developing pericardial delivery approaches to broaden patient access to cardiac reprogramming (Fig. 3D) [121]. While early structural milestones demonstrated the technical feasibility of this route by delivering therapeutic biomaterials less-invasively into the pericardial cavity rather than targeting cardiac lineage conversion directly, contemporary adaptations leverage catheter-based technologies to deliver reprogramming factors directly to the heart without open-chest surgery, offering advantages such as reduced procedural risk and shorter recovery time.

Notable advances include catheter-based systems integrated with imaging modalities such as intracardiac echocardiography and electroanatomical mapping [122]. These systems enable real-time visualization and precise targeting of specific myocardial regions, potentially enhancing the efficiency of reprogramming factor delivery. In parallel, the use of biocompatible hydrogels [123,124] and controlled-release systems [125,126] for pericardial administration is being explored to achieve sustained factor release over extended periods.

Despite these advances, several challenges remain, including ensuring uniform distribution of reprogramming factors, optimizing delivery parameters, and engineering catheters capable of accessing all regions of the heart [127,128]. To cross the critical bridge from rodent models to human application, recent pivotal studies have utilized porcine MI models to assess the translational feasibility of this route [129]. Meticulous long-term safety evaluations in these swine settings have demonstrated that catheter-mediated pericardial hydrogel delivery successfully mitigates severe procedural surgical risks and significantly improves therapeutic retention within the ischemic zone [130]. Crucially, these large-animal studies reported notable recovery in cardiac function indicators—specifically raising the left ventricular ejection fraction (EF) and fractional shortening (FS)—while successfully demonstrating long-term electrical safety by suppressing ventricular arrhythmias or ectopic electrical shocks [131]. The safety and efficacy of these approaches require rigorous evaluation in preclinical models before translation to human trials. Nevertheless, pericardial delivery systems could overcome a major barrier to clinical cardiac reprogramming, making this therapy more feasible for patients with heart failure or other cardiac disorders.

### 3.3 Translational balance and technical bottlenecks of factor delivery platforms

A multi-dimensional evaluation of current gene delivery systems reveals a fundamental trade-off between genomic safety and transduction competence that dictates their clinical translation potential (Table 2). Viral vectors, specifically SeV and AAV, remain the benchmark for direct reprogramming efficiency due to their evolutionary optimization for cell entry and sustained factor expression duration [119]. However, their translational feasibility is severely bottlenecked by strict cargo size constraints—particularly for AAV (∼4.7 kb), which complicates the packaging of multi-cistronic transcription factor cocktails—and the persistent risk of host immune clearance or rare insertional mutagenesis [132]. Conversely, non-viral synthetic and biological platforms sacrifice absolute, single-dose delivery efficiency to achieve superior genomic safety profiles and highly scalable manufacturability [107]. LNP-modRNA systems eliminate any integration-associated risks and offer unlimited cargo packaging capacity, yet their rapid enzymatic clearance and short intracellular half-life demand optimization of cell-type specific promoters or repeat-dosing regimens to achieve full structural and electrophysiological maturation in CFs [119]. While biological nanovesicles like EVs present an ideal paradigm for immune evasion and cell-type specificity via surface peptide engineering, their clinical translation is heavily restricted by low cargo encapsulation yields and the lack of high-throughput, cGMP-compliant isolation frameworks to overcome biological batch-to-batch heterogeneity. Ultimately, Table 2 provides a comprehensive overview that rationalizes these intricate and overlapping parameters; however, achieving high-fidelity cardiac reprogramming requires not only an optimized delivery vehicle but also its synergistic integration with cellular microenvironmental cues, as detailed in the subsequent section.

**Table 2:** Comprehensive comparison of delivery platforms for direct cardiac reprogramming.

Delivery System	Specific Platform	Cargo capacity	Duration of expression	Cell-type specificity	Delivery/reprogramming efficiency	Immunogenicity/Toxicity	Off-target risks/genomic integration	Manufacturability/scalability	Clinical/regulatory feasibility	Key advantages	Major limitations
**Viral Vectors (integration-free)**	**Sendai virus (SeV)**	High (polycistronic cocktail carriage)	Sustained (robust factor expression)	Moderate (Efficiently targets proliferating CFs)	High (efficient and rapid generation)	Moderate (requires mitigating potential immune responses)	None (does not integrate into host genome, non-integrating)	Moderate (requires optimizing manufacturing processes)	Moderate (pending clinical translation validation)	Efficient and rapid integration-free factor expression; proven cardiac function improvement *in vivo*	Potential to trigger host immune responses; complex manufacturing optimization
	**Adeno-associated virus (AAV)**	Limited (standard packaging limits)	Persistent (stable transgene expression dynamics)	Moderate to high (engineered variants like AAV-DJ target CFs)	Moderate to high (efficient gene delivery to CFs)	Low (valued for immunogenicity)	None (non-integrating nature)	High (attractive for clinical gene therapy)	High (widely used in clinical gene therapy applications)	Low immunogenicity, non-integrating nature, and efficient fibroblast gene delivery	Safety profiles and transgene expression dynamics require further long-term evaluation
**Synthetic nanoparticles**	**Gold nanoparticles (AuNPs)**	High (high surface-to-volume ratio for cargo binding)	Short/transient (protects and carries cargo transiently)	Low (passive unless modified with surface targeting)	Moderate (demonstrated improved *in vivo* efficiency over old methods)	Low (valued for its non-toxic nature)	None (non-viral alternative, completely non-integrating)	High (ease of chemical functionalization)	Moderate (paves the way for safer clinical applications)	Non-toxic nature, high surface-to-volume ratio, and ease of functionalization for cargo protection	Long-term biodistribution of metallic particles and surface charge control challenges
	**Lipid nanoparticles (LNPs)**	High (flexible encapsulation of large modRNA cocktails)	Short/transient (transient, dose-controllable kinetics)	Low to moderate (naturally liver-dominant; re-routed via SORT technology)	High (robust *in vivo* delivery to post-ischemic tissue)	Moderate (elevated doses can spark transient innate pathway activation)	None (zero risk of integration-associated insertional mutagenesis)	Very high (outstanding scalability from global vaccine deployment)	High (clinically proven success in global deployment frameworks)	High transfection efficiency, scalable production, and zero genomic integration risk	Brief intracellular half-life requires repeated dosing; risks localized immunogenicity at high doses
**Biological nanovesicles**	**Extracellular vesicles (EVs)**	Moderate (constrained by natural vesicle limits)	Sustained (loaded modRNA/miRNA enables sustained factor expression)	High (surface-engineered with cardiac-targeting peptides)	High (achieves targeted delivery in damaged myocardium)	Very low (bypasses immune surveillance, minimizes immunogenicity)	None (safe non-viral biological platform)	Low (hurdles in standardizing cargo-loading efficiency)	Moderate (challenges in ensuring batch-to-batch consistency)	Superior cargo stability, targeted delivery, low immunogenicity, and ability to evade immune surveillance	Biological variability of donor cells affects batch-to-batch consistency and cargo-loading scalability
**Naked nucleic acids**	**Modified mRNA (modRNA)**	High (no packaging size restrictions for cocktails)	Short/transient (transient expression profiles)	Low (requires promoter-based or vehicle targeting to avoid off-targets)	Moderate to high (enhances cardiac function and reduces fibrosis)	Low (chemically modified to minimize acute immune rejection)	None (no integration footprints, transient action)	High (pure chemical synthesis alternative)	High (innovative approach drawing considerable attention)	Enhances cardiac function and reduces fibrosis without genomic alterations or long-term safety legacy	Limited maturity of iCMs; potential long-term consequences under study
**Administration route**	**Pericardial delivery**	N/A (applies to all vehicles combined with hydrogels)	Controllable (sustained via bioresponsive hydrogels)	Moderate to high (enhances agent retention in infarcted tissue)	Moderate to high (enhances agent retention in infarcted tissue)	Low (less invasive, reduces severe procedural surgical risks)	N/A (vehicle-dependent)	Moderate (leverages catheter-based technologies and real-time imaging)	Moderate to high (broadens patient access compared to intramyocardial routes)	Less-invasive approach, reduced procedural risk, shorter recovery time, and enhanced sustained release	Challenges in ensuring uniform distribution and engineering catheters to access all heart regions

## 4 New strategies for environmental modification

### 4.1 Immune-modulating environment

The immune system plays a complex role in cardiac reprogramming, acting both as a facilitator and a potential barrier to the process. Rather than exerting generic inflammatory effects, specific microenvironmental components establish precise molecular barriers; for instance, recent breakthroughs reveal that blocking the nuclear receptor Nr4a3 actively unlocks the cellular senescence barrier to robustly accelerate direct conversion [133]. Recent studies have revealed that the inflammatory microenvironment profoundly influences the conversion of CFs into CMs, where host-derived chronic pathways-specifically dominated by CXCL4 and interleukin-1α (IL-1α) signaling axes-operate alongside the interferon-α/β receptor (IFN-IFNAR)-p-STAT1 cascade to actively suppress downstream cardiogenic factor transcription [53,134,135].

**Fig. 4:**
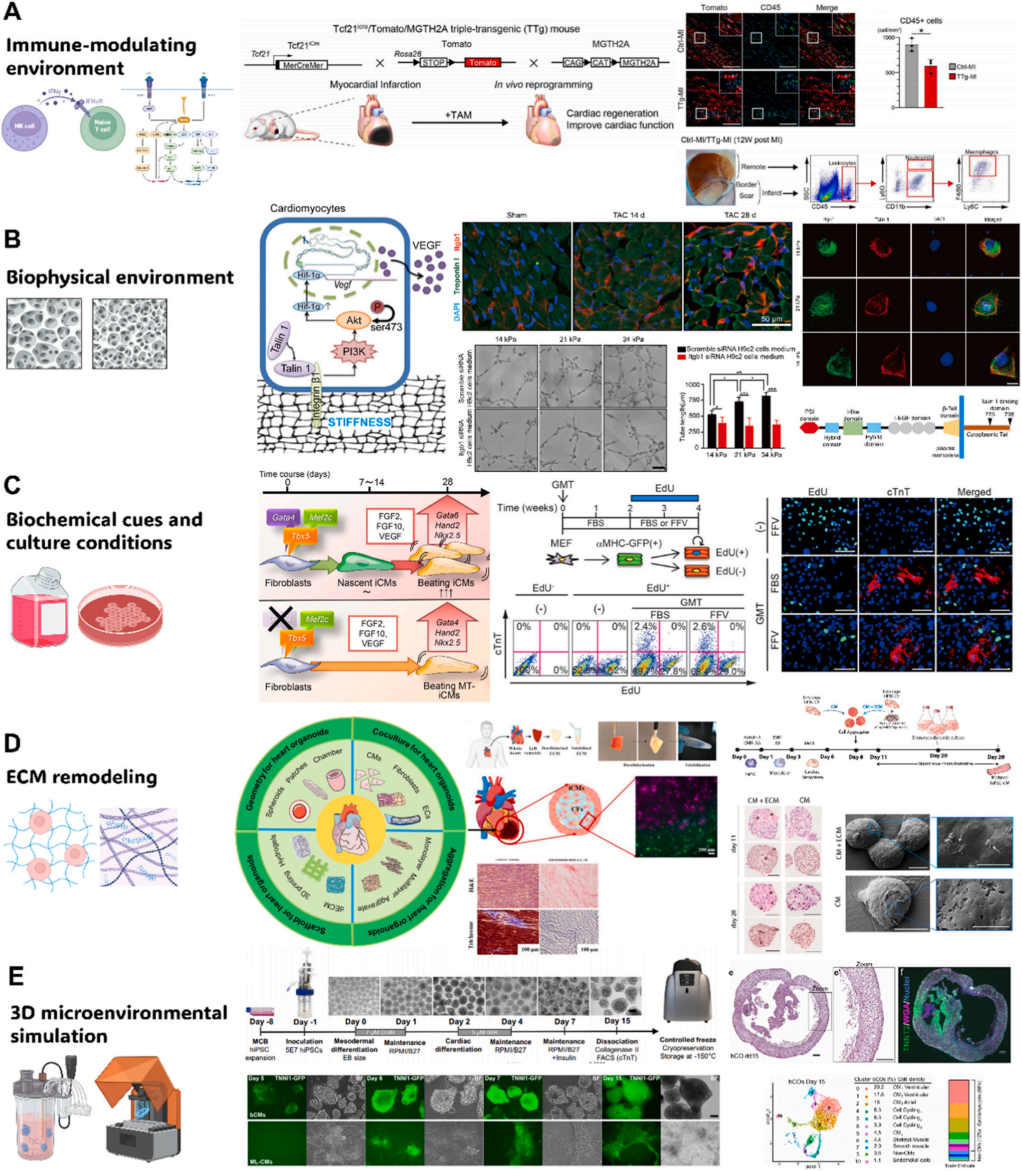
Environmental modifications enhancing cardiac reprogramming. (A) Immune-modulated microenvironment. Cardiac reprogramming attenuates chronic inflammation and reduces CCR2^+^ macrophage infiltration in infarcted hearts. Data are obtained and reused from Abe et al., Cardiac reprogramming reduces inflammatory macrophages and improves cardiac function in chronic myocardial infarction. *Biochem. Biophys. Res. Commun.* 2024, 690, 149272 with permission. (B) ECM-based biomechanical regulation. Extracellular matrix (ECM) stiffness critically influences cellular commitment and direct cardiac reprogramming efficiency. Data are obtained and reused from Shen J et al., Increased myocardial stiffness activates cardiac microvascular endothelial cell via VEGF paracrine signaling in cardiac hypertrophy. *J Mol Cell Cardiol.* 2018, 122, 140–151 with permission. (C) Biochemical optimization. Growth factors for fibroblasts and endothelial cells establish optimal culture conditions for cardiac reprogramming efficiency. Data are obtained and reused from Yamakawa et al., Fibroblast growth factors and vascular endothelial growth factor promote cardiac reprogramming under defined conditions. *Stem Cell Rep*. 2015, 5(6), 1128-1142; Korf-Klingebiel et al., Myeloid-derived growth factor protects against pressure overload–induced heart failure by preserving sarco/endoplasmic reticulum Ca2+-ATPase expression in cardiomyocytes. *Circulation*. 2021, 144(15), 1227-1240 with permission. (D) Cardiac ECM remodeling. Heart-derived ECM significantly enhances direct cardiac reprogramming efficacy. Data are obtained and reused from Wang Y et al., Model construction and clinical therapeutic potential of engineered cardiac organoids for cardiovascular diseases. *Biomater Transl.* 2024, 5, 337; Almeida HV et al., Human extracellular-matrix functionalization of 3D hiPSC-based cardiac tissues improves cardiomyocyte maturation. *ACS Appl Bio Mater.* 2021, 4, 1888–1899 under a Creative Commons Attribution 4.0 International License with permission. (E) 3D microenvironmental engineering. Suspension culture systems optimize cardiogenesis in iPSC-derived cardiac organoids. Data are obtained and reused from Prondzynski et al., Efficient and reproducible generation of human iPSC-derived cardiomyocytes and cardiac organoids in stirred suspension systems. *Nat. Commun*. 2024, 15(1), 5929 under a Creative Commons Attribution 4.0 International License with permission.

Importantly, the role of inflammation differs between murine and human systems [136]. While murine studies suggest that inflammation acts as a barrier to reprogramming [137], investigations in human fibroblasts indicate that specific immune signaling pathways—including TLR3, NFKB1, and PTGS2—may be essential for successful cardiac reprogramming (Fig. 4A) [23,138]. Anti-inflammatory compounds and chemokine receptor inhibitors have shown promise in enhancing reprogramming efficiency.

Understanding the delicate balance between immune activation and suppression remains critical to advancing cardiac reprogramming strategies. The temporal dynamics of immune responses—characterized by inflammation, proliferation and maturation phases—directly influence tissue repair and reprogramming potential [139,140]. Continued research focused on precise immune modulation will be essential for translating these insights into clinically viable therapies for heart failure and cardiac regeneration. From a translational perspective, this delicate immunological balance is heavily tested in preclinical large-animal models; for instance, in porcine MI injury settings, the temporal transition from pro-inflammatory M1 macrophages to reparative M2 phenotypes directly governs the *in vivo* survival of delivered factors [[141], [142], [143]]. While rodent studies provide basic mechanistic screening for immune barriers, validating how chronic chemokine cascades affect factor retention in human-scale fibrotic tissue remains a mandatory intermediate requirement prior to human trials.

### 4.2 Biophysical environment

The mechanical properties of the substrate play a crucial role in cardiac reprogramming [144]. Softer matrices mimicking native myocardium (∼10 kPa) enhance reprogramming efficiency by inhibiting key signaling pathways, including integrin (Fig. 4B) [145,146], Rho/ROCK [147,148], actomyosin [149,150], and YAP/TAZ [151,152]. This suppression reduces fibroblast-specific gene expression, facilitating cellular conversion towards a CM phenotype.

Surface modifications—particularly microgrooved substrates—have emerged as powerful tools to improve reprogramming outcomes. These modifications not only increase reprogramming efficiency but also promote the organization of sarcomeric structures in induced CMs [153,154]. By providing alignment cues and activating pro-reprogramming factors such as histone H3 acetylation and myocardin sumoylation, these engineered surfaces guide spatial cell organization [155,156].

The intricate interplay between the biophysical environment and cellular reprogramming offers promising strategies for cardiac regeneration. By carefully engineering substrate properties and surface topography, researchers can create more conducive conditions for efficient and functional cardiac reprogramming, potentially advancing therapeutic approaches for heart repair and regeneration. However, it is critical to note that the vast majority of these biophysical milestones—such as substrate softening (∼10 kPa) or microgroove-mediated alignment—remain strictly confined to two-dimensional *in vitro* screening environments [63,64]. Translating these biophysical cues to actively beating, massive human or large-animal hearts *in vivo* remains a major structural bottleneck, as actively expanding fibrotic scars cannot be easily re-engineered mechanically post-infarction.

### 4.3 Biochemical cues and culture conditions

Culture conditions supplemented with growth factors and cytokines play a pivotal role in promoting *in vitro* cardiac reprogramming. These environmental cues significantly enhance the generation of functionally mature iCMs by providing essential signaling molecules that drive the reprogramming process [157]. Factors such as FGF2, FGF10 and VEGF have been shown to improve reprogramming efficiency and iCM maturation, highlighting the importance of recreating the complex signaling environment of the native myocardium [158].

Oxygen levels in the microenvironment have emerged as a crucial determinant of reprogramming success. Short-term pretreatment of fibroblasts under low oxygen (hypoxic) conditions significantly improves the efficiency of GMT-based cardiac reprogramming [159]. This hypoxic preconditioning actively triggers metabolic shifts and chromatin accessibility rearrangements, priming cells for more efficient reprogramming. Thus, oxygen availability is a critical environmental cue influencing both fate conversion and iCM maturation.

Optimizing culture media composition and substrate properties can further enhance the reprogramming process. For example, the use of defined serum-free media supplemented with specific growth factors can reduce variability and improve reproducibility in reprogramming experiments (Fig. 4C) [160]. Additionally, three-dimensional culture systems such as hydrogels or scaffolds that mimic the native cardiac extracellular matrix (ECM) have shown promise in creating a more physiologically relevant microenvironment for iCM generation and maturation [161,162]. These advanced culture systems provide biochemical and biophysical cues that synergistically promote cardiac reprogramming and functional maturation of iCMs. Concurrently, while hypoxic preconditioning and serum-free growth factor cocktails (FGF/VEGF) effectively prime rodent fibroblasts, their application to human clinical tracks is limited [163,164]. Systemic or prolonged delivery of such biochemical cues in large-animal configurations faces brief *in vivo* half-lives and risks inducing unintended off-target oncogenesis or untargeted systemic angiogenesis, restricting this strategic module primarily to controlled, *ex vivo* bioengineered tissue processing [165,166].

### 4.4 Extracellular matrix remodeling

Decellularized heart extracellular matrix (ECM) has emerged as a powerful three-dimensional biomaterial for reconstructing the microenvironment in cardiac reprogramming [167,168]. This native ECM provides a complex array of biochemical and biophysical cues that closely mimic the natural cardiac milieu, enhancing both chemical reprogramming efficiency and CM maturation. Studies have shown that reprogrammed CMs cultured within this ECM exhibit significantly elevated expression of cardiac-specific markers, improved sarcomeric organization, and enhanced electrophysiological properties compared to those cultured on conventional substrates.

The therapeutic potential of CMs reprogrammed within a three-dimensional heart ECM has been investigated in animal models of MI, particularly in rats (Fig. 4D) [169,170]. These studies demonstrate improved cell survival, integration, and functional outcomes when reprogrammed cells are delivered within an ECM scaffold. The ECM not only provides structural support but also facilitates cell–matrix interactions that are crucial for cell survival and function in the hostile environment of the infarcted myocardium. Importantly, decellularized ECM (dECM)-based microenvironmental remodeling represents one of the few strategies that has successfully transitioned from rodent models to large-animal preclinical validation [171]. Preclinical studies utilizing porcine MI settings have verified that injectables combined with tissue-specific dECM hydrogels significantly enhance local therapeutic retention and drive long-term safety, establishing a functional bridge toward clinical heart failure frameworks [172]. This approach represents a promising strategy to enhance the efficacy of cardiac reprogramming therapies in clinical applications, potentially improving outcomes in heart failure treatment.

### 4.5 Microenvironmental simulation using 3D technology

Recent advancements in 3D bioprinting and organoid technology have opened new avenues for mimicking the complex cardiac microenvironment, potentially enhancing the efficiency and quality of direct cardiac reprogramming [[173], [174], [175]]. 3D bioprinting enables the precise deposition of cells, biomaterials and growth factors in a spatially controlled manner, creating structures that closely resemble native cardiac tissue architecture [176,177]. This technology facilitates the fabrication of cardiac-specific ECMs with tailored mechanical properties and biochemical compositions, providing a more physiologically relevant environment for cell reprogramming. Moreover, 3D bioprinted constructs can incorporate multiple cell types and biomolecules in defined spatial arrangements, allowing study of cell–cell and cell–matrix interactions during the reprogramming process [178,179].

Cardiac organoids, by contrast, offer a powerful platform for recapitulating the developmental processes and cellular interactions of the heart *in vitro* [180,181]. These self-organizing, three-dimensional structures can be generated from pluripotent stem cells or directly reprogrammed cells, providing a unique model for studying cardiac development, disease and regeneration (Fig. 4E) [182]. By incorporating reprogramming factors into organoid culture systems, researchers can investigate the influence of tissue-specific cues on the efficiency and fidelity of cardiac reprogramming [183,184]. Furthermore, cardiac organoids serve as valuable tools for drug screening and toxicity testing, potentially accelerating the translation of direct cardiac reprogramming therapies to clinical applications [185]. The integration of 3D bioprinting and organoid technologies in cardiac reprogramming research holds great promise for optimizing protocols and developing more effective regenerative therapies for heart failure. Crucially, while delivery platforms and biophysical cues are heavily rodent-centric, 3D organoid simulation serves as the premier human-centric translational check-point [186]. By integrating reprogramming cascades directly into 3D human ventricular organoids, researchers can investigate human-specific chromatin accessibility and electrophysiological integration within a human genetic background [167]. This multi-cellular simulation bypasses the historical pitfalls of extrapolating murine data, providing an invaluable benchmarking platform to address human-specific efficiency barriers and long-term genomic stability before progressing to actual patient clinical entry.

### 4.6 Translational matrix and functional trade-offs in microenvironmental engineering

To maximize the therapeutic efficacy of direct cardiac reprogramming, the strategic integration of the multifaceted environmental cues described above must be systematically contextualized (Table 3). A cross-examination of these developmental, physical, and cellular niches reveals that each bioengineering module operates at a distinct stage of translational maturity [166]. Biochemical cues, media standardization, and biophysical substrate tuning serve as highly efficient, standardized platforms for upstream *in vitro* screening and initial fate-commitment acceleration; however, their active clinical transition is heavily restricted by brief *in vivo* growth factor half-lives, off-target systemic oncogenesis risks, and the technical impossibility of mechanically re-engineering rigid, beating infarcted ventricular scars in live patients [163]. Conversely, complex biomimetic strategies, such as tissue-specific dECM remodeling, offer robust, clinically validated performance in preclinical large-animal models by directly facilitating cell-matrix signaling, host electrophysiological integration, and long-term therapeutic retention within aggressive ischemic zones [172]. Concurrently, multi-cellular 3D organoid simulation is steadily establishing itself as the premier human-centric benchmarking platform to investigate human-specific chromatin accessibility landscapes and long-term genomic stability without the ethical and physiological pitfalls of over-extrapolating rodent milestones [167,186]. Ultimately, as summarized in Table 3, overcoming chronic cross-species immunological discrepancies and establishing fine, temporal orchestration over these biological, chemical, and physical parameters represent the definitive milestones for moving advanced microenvironmental engineering from laboratory settings into personalized cardiovascular medicine.

**Table 3:** Summary of microenvironmental engineering strategies for enhanced cardiac reprogramming.

Strategy category	Proposed mechanisms	Core advantages/pros	Major limitations/cons	Translational status
**Immune-modulating environment**	• Suppresses the IFN-IFNAR-p-STAT1 pathway driven by inflammatory macrophages • Activates human-specific TLR3 and NF-κB cascades required for chromatin opening	• Resolves the hostile, chronic fibrotic niche • Mitigates early cell death and boosts initial fate conversion	• Severe cross-species discrepancies (inflammation hinders mice but aids human models) • Fine temporal control is highly complex	Preclinical validation (effectively demonstrated in rodent post-MI models via combination therapies)
**Biophysical & mechanical tuning**	• Soft matrices (∼10 kPa) inhibit pro-fibrotic Integrin/ROCK/YAP/TAZ mechanosignaling • Microgrooved topographies induce histone H3 acetylation and structural alignment	• Suppresses competing fibroblast-specific gene programs • Accelerates downstream sarcomeric organization and maturation	• Difficult to precisely manipulate physical tissue stiffness in live *in vivo* hearts • Over-reliance on 2D culture setups	*In vitro* benchmarking/early *in vivo* (mechanisms well-defined *in vitro*; translates via epicardial patches)
**Biochemical cues & hypoxic preconditioning**	• Growth factor cocktails (FGF2, FGF10, VEGF) stimulate endogenous angiogenic pathways • Hypoxia triggers HIF-1α to prime metabolic transitions toward cardiac lineages	• Promotes robust cell proliferation and functional iCM maturation • Standardizes protocols under defined, serum-free conditions	• Growth factors have short half-lives *in vivo* • Systemic delivery risks inducing unintended tumorigenesis or off-target angiogenesis	*In vitro* standardized/preclinical (serum-free media and pre-conditioning widely used in laboratory settings)
**Extracellular matrix (ECM) remodeling**	• Decellularized heart matrix (dECM) provides a native 3D biochemical scaffold • Enhances crucial cell-matrix signaling via natural integrin bindings	• Substantially improves electrophysiological integration and tissue maturation • Enhances long-term cell survival in infarcted tissue	• Biological variations in donor dECM lots • Potential risks of pathogen transmission or minor immunogenic reactions	Preclinical validation (successfully validated in rat and large-animal MI models via injectables)
**3D technology & organoid simulation**	• 3D bioprinting organizes cells and structural biomaterials with spatial precision • 3D suspension organoids self-assemble to mimic native intercellular cross-talk	• Accurately recapitulates multi-cellular organization and 3D native tissue stiffness • Serves as a predictive model for drug screening and disease modeling	• Vascularization bottlenecks limit structural scale-up • High technical complexity and cost constraints	In vitro screening/translational models (emerging as a premier platform for validating human-specific translation)

## 5 Future directions

**Fig. 5:**
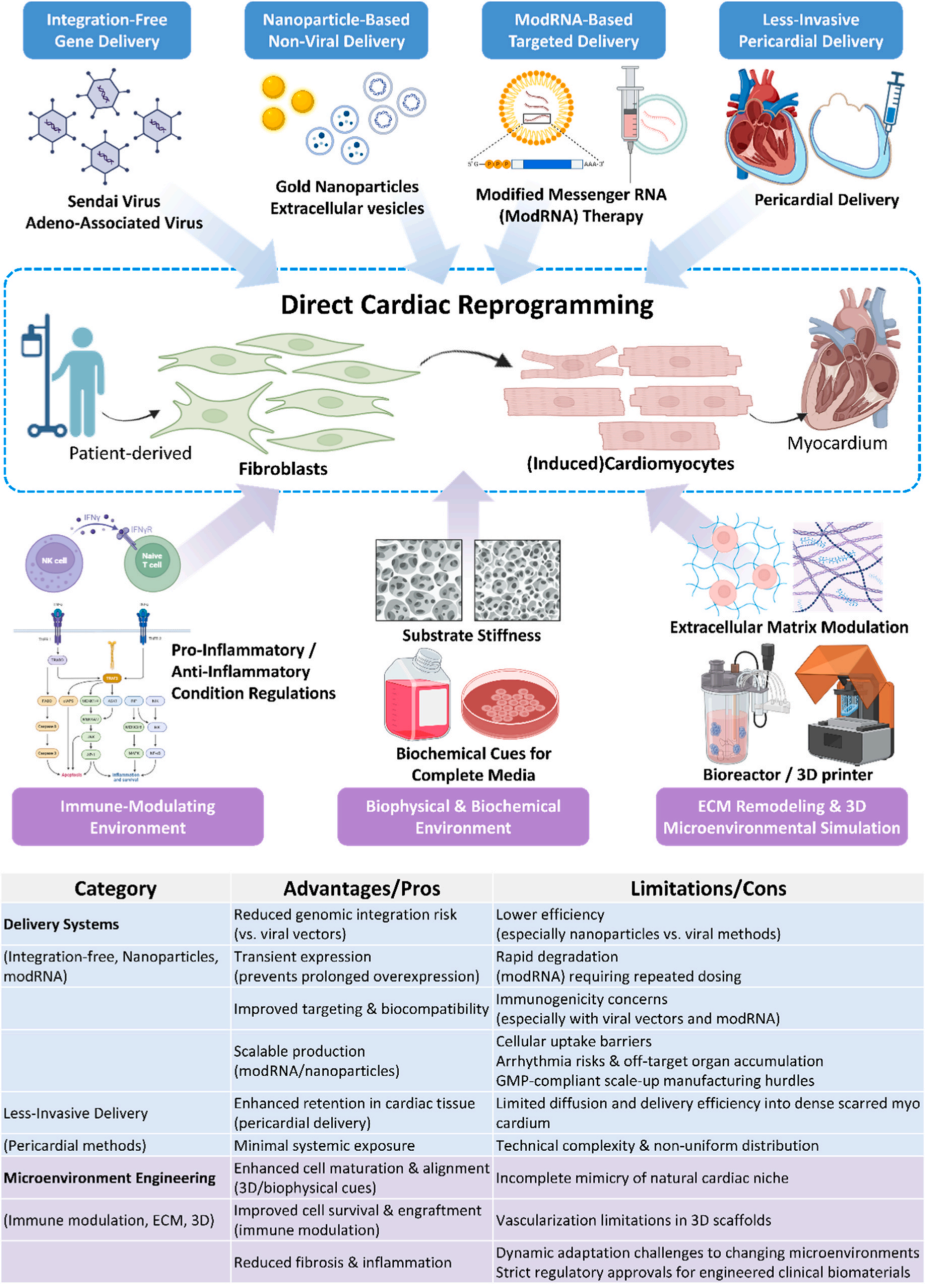
Newly developed approaches for enhanced direct cardiac reprogramming. This schematic compares advanced strategies to improve direct cardiac reprogramming. Innovations include delivery system advancements—such as integration-free gene delivery, nanoparticle-based non-viral delivery, modRNA-based targeted delivery, and less-invasive pericardial delivery—and microenvironment engineering, including immune modulation, biophysical and biochemical environment optimization, and ECM remodeling with 3D microenvironmental simulation. These approaches complement existing direct conversion strategies and demonstrate synergistic potential for myocardial regeneration. A comparative table summarizes the advantages and limitations of each method, highlighting critical considerations for clinical translation.

The future trajectory of direct cardiac reprogramming for heart failure treatment hinges on addressing its multifaceted challenges and exploring innovative approaches to enhance its therapeutic potential (Fig. 5). A critical focus lies in achieving an optimal balance between reprogramming efficiency and functional completeness, ensuring the generation of fully functional CMs while maintaining high conversion rates. This delicate equilibrium will require the development of sophisticated techniques for precise control and monitoring of each stage of the reprogramming process. Concurrently, elucidating the intricate molecular mechanisms governing cell fate determination through cutting-edge methodologies such as single-cell transcriptomics and spatial genomics will be pivotal in optimizing reprogramming protocols [187,188] These insights could enable researchers to fine-tune the expression of key transcription factors and signaling molecules, potentially uncovering novel reprogramming factors or factor combinations.

### 5.1 Overcoming species-specific barriers and modeling human-centric translation

Overcoming the profound species-specific barriers between murine models and human physiology remains a critical hurdle for future clinical success. Given that human CFs (hCFs) exhibit distinct transcriptional profiles, restrictive epigenetic landscapes, and unique immunological responses compared to their murine counterparts, relying solely on rodent data risks translational failure [189,190]. Therefore, rigorous validation using human-derived primary cells, hiPSC-derived cardiac lineages, and multi-cellular 3D human cardiac organoid models must be implemented as a mandatory benchmarking step to accurately predict efficacy and safety in clinical settings [167,186]. This challenge underscores the need for comprehensive comparative studies between murine and human cellular responses to reprogramming stimuli, potentially leading to the identification of human-specific factors or pathways that may enhance reprogramming efficiency in clinically relevant cell populations [191,192]. Furthermore, the consistent observation that *in vivo* reprogramming often yields superior results compared to *in vitro* approaches highlights the critical role of the native cardiac microenvironment [193,194]. Future research should focus on deciphering the complex interplay between reprogramming factors and the native cardiac milieu, with the ultimate goal of recapitulating these native conditions *in vitro* through advanced 3D culture systems, organoids, or bioengineered scaffolds to ensure high-fidelity lineage conversion.

### 5.2 Next-generation epigenetic tools and precision delivery frontiers

To transcend the limitations of conventional factor delivery, the integration of emerging genetic tools and precision engineering represents a promising frontier for the field. Precise epigenetic control is rapidly shifting the paradigm, with CRISPR-Cas9-based epigenetic editing technologies offering unprecedented opportunities to modulate chromatin structure, histone modifications, and DNA methylation patterns without altering the primary sequence [195,196]. These programmable multiplexed approaches could significantly enhance both the efficiency and fidelity of the reprogramming process by selectively opening tightly sealed human cardiac loci [197]. In parallel, the development of advanced delivery systems utilizing targeted nanoparticles, exosomes or LNP-mediated mRNA technologies could revolutionize the administration of reprogramming factors [198]. By optimizing these non-viral vehicles to improve cargo stability, cellular uptake, and spatiotemporal control of factor expression, researchers can effectively mitigate the safety concerns, such as chronic immunogenicity or insertional mutagenesis, historically associated with viral vector-based approaches, thereby establishing a chemically defined and highly controllable translational framework.

### 5.3 Preclinical roadmaps, technical bottlenecks, and clinical entry

As the field steadily advances from laboratory proof-of-concept towards clinical validation, a structured roadmap resolving scalable manufacturing and deep-seated technical bottlenecks must be established. Industrially, the mass production of non-viral vectors and modRNA cocktails under clinically compliant cGMP conditions demands strict standardization of microfluidic formulations to ensure batch-to-batch consistency and resolve chemistry, manufacturing, and control (CMC) hurdles [199]. Biologically, several interconnected barriers remain to be systematically addressed, most notably incomplete CM maturation, where induced cells retain an embryonic phenotype. Overcoming this requires temporal microenvironmental synchronization to eliminate electrophysiological unsaturation and its associated arrhythmogenic risks [167]. Immature iCMs frequently display unstable resting potentials and I_K1_ potassium current deficits, which can prompt ectopic pacing or lethal ventricular arrhythmias upon integration [200]. Future frameworks must enforce synchronous coupling via gap-junction proteins like Connexin 43 to guarantee electrical safety within the host myocardium, while continuously tracking long-term genomic stability to rule out epigenetic reversion or tumorigenicity [201]. Furthermore, verifying these safety profiles necessitates the meticulous design of preclinical large-animal models, such as telemetry-implanted porcine or non-human primate MI settings, to monitor dose-dependent organ retention and electrical stability under realistic human-scale geometries [202]. Finally, exploring the potential of combining direct reprogramming with other advanced regenerative approaches, such as tissue engineering or cell therapy, could yield profound synergistic effects, enhancing overall cell engraftment and structural recovery in the hostile infarcted environment [203]. By systematically addressing these interconnected technical and regulatory challenges under standardized production standards, direct cardiac reprogramming could solidify its status as a paradigm-shifting approach in personalized medicine, offering tailored, patient-specific strategies and new therapeutic hope for millions of individuals suffering from debilitating heart failure globally.

## 6 Conclusions

Direct cardiac reprogramming has rapidly evolved from a conceptual breakthrough to a promising therapeutic strategy for heart failure, addressing the urgent need for regenerative solutions in a field where conventional treatments remain palliative or are constrained by donor shortages. Over the past decade, significant strides have been made in optimizing reprogramming factor cocktails, refining delivery systems, and engineering the cardiac microenvironment to enhance overall efficiency, safety, and clinical applicability of this approach. Innovations in integration-free gene delivery—such as Sendai virus and adeno-associated virus vectors—have mitigated concerns about genomic integration and off-target effects, while nanoparticle- and modRNA-based systems have opened new avenues for transient, non-toxic, and highly targeted reprogramming.

Simultaneously, advances in pericardial and minimally invasive delivery methods have improved the feasibility and therapeutic retention of therapeutic agents in infarcted cardiac tissue, moving the field closer to widespread clinical application. Microenvironmental modulation—encompassing immune regulation, substrate stiffness optimization, and the use of 3D-bioprinted matrices—has proven essential for both the efficiency and maturation of induced CMs. These strategies not only facilitate the reprogramming process but also address challenges related to engraftment, functional integration, and long-term stability within the hostile milieu of the injured heart.

Preclinical studies in large-animal models have validated the translational potential of these innovations, demonstrating substantial improvements in cardiac function, scar reduction, and cell engraftment. As a result, direct cardiac reprogramming is steadily advancing toward clinical validation, offering a long-term prospect of redefining the therapeutic landscape for the more than 64 million individuals affected by heart failure globally. Despite this progress, several critical challenges remain. Achieving consistent ventricular subtype specification, ensuring long-term genomic and functional stability, and overcoming immune compatibility barriers are essential for safe and effective clinical translation.

The integration of advanced technologies—such as single-cell omics, CRISPR-based epigenetic editing, and 3D tissue engineering—will be pivotal to overcoming these hurdles and refining reprogramming protocols for human application. In summary, the convergence of optimized delivery systems, microenvironmental engineering, and mechanistic insights has transformed direct cardiac reprogramming from a laboratory curiosity into a viable candidate for regenerative heart failure therapy. Continued interdisciplinary collaboration and rigorous translational research will be crucial for realizing the full potential of this paradigm-shifting approach, ultimately paving the way for personalized, durable, and restorative treatments in cardiovascular medicine.

## CRediT authorship contribution statement

**Boram Son:** Conceptualization, Data curation, Investigation, Methodology, Project administration, Resources, Supervision, Visualization, Writing – original draft, Writing – review & editing. **Gwang Yeol Park:** Writing – original draft, Writing – review & editing. **Ildoo Jeong:** Writing – original draft, Writing – review & editing. **Hwanhui Kim:** Writing – original draft, Writing – review & editing. **Jinkyu Lee:** Writing – original draft, Writing – review & editing. **Jinmyoung Joo:** Writing – original draft, Writing – review & editing. **Wonhwa Lee:** Funding acquisition, Project administration, Supervision, Visualization, Writing – original draft, Writing – review & editing. **Hee Ho Park:** Conceptualization, Data curation, Funding acquisition, Investigation, Methodology, Project administration, Resources, Supervision.

## Declaration of competing interest

The authors declare that they have no known competing financial interests or personal relationships that could have appeared to influence the work reported in this paper.

## Data Availability

Data will be made available on request.
